# Diagnosis and operatory treatment of the patients with failed back surgery caused by herniated disk relapse

**Published:** 2014

**Authors:** A Bodiu

**Affiliations:** * Institute of Neurology and Neurosurgery, Chisinau, Republic of Moldova

**Keywords:** failed back surgery syndrome, repeated discectomy, transforaminal lumbar interbody fusion (TLIF), recurrent lumbar disc herniation, repeated laminotomy

## Abstract

**The object of study:** Analysis of surgical treatment results in patients with recurrent lumbar disc herniation by transforaminal lumbar interbody fusion (TLIF) and repeated laminotomy and discectomy for the improvement of pain and disability.

**Material and methods:** Data analysis was performed on a complex diagnosis and treatment of 56 patients with recurrent lumbar disc herniation who had previously underwent 1-3 lumbar disc surgeries.

An MRI investigation with paramagnetic contrast agent (gadolinium) was used for the diagnosis and differentiation of epidural fibrosis, and a dynamic lateral X-ray investigation was carried out for the identification of segmental instability.

The evolution period after the previous surgery was between 1 and 3 years after the index surgery.

Pain expression degree and dynamics were assessed with the pain visual analog scale (VAS) in early and late postoperative periods. Postoperative success was assessed by using a modified MacNab scale. The follow-up recording period after the last operation was of at least 1 year, ranging from 1 to 4 years.

**Results:** The surgical treatment was effective in most cases, recording a reduction in pain expression level from 7.2 - 7.7 points on the VAS scale to 1.7 - 2.1 in the early period and 2.2 – 2.6 in the late period (1 year).

Repeated surgery was effective in 21 of 30 (70%) cases who underwent decompression surgery without fusion and in 20 of 26 (76.9%) cases who underwent repeated surgery with transforaminal lumbar interbody fusion (TLIF). Overall, postoperative success was assessed by using a modified MacNab scale.

**Conclusion:** Repeated surgery is a viable option for patients who have clinical manifestations of recurrent disc herniation. Investigation with contrast agent by MRI allows differentiating disk herniation recurrences from epidural fibrosis.

Supplementing repeated discectomies and decompression with intervertebral transforaminal fusion provide superior clinical outcomes, especially in patients with clinical and radiological signs of lumbar segment instability.

The unsuccessful surgeries’ rate in the back surgery treatment is between 10 and 33% [**3**–**5**,**7**,**11**–**13**]. Together with the raise in the number of surgeries, the number of patients who need repeated surgeries has also raised. This has led to the appearance of the notion of ”Failed Back Surgery Syndrome (FBSS)”, which is at present considered more a special disease than a postoperatory complication [**3**–**6**,**12**].

The largest group of patients with FBSS is represented by the ones who underwent surgery due to herniated lumbar disc by a posterior approach. There are many causes for the appearance of repeated neurovascular compression, and, among them, the following can be mentioned: epidural fibrosis, segmental instability, segmental stenosis and disc recurrences. Most often a combination of these causes can be noticed [**1**,**2**,**4**–**6**,**14**,**15**]. The reasons most frequently met for a repeated surgery are the following: level error, ipsilateral or contralateral disc recurrence, insufficient decompression, secondary stenosis through peridural fibrosis, instability of the operated segment [**2**,**3**,**5**,**7**,**9**,**10**,**11**].

The most frequent manifestation of a disc recurrence is pain, which can be similar to the one that served as a reason for the first surgery, and then, an ipsilateral disc recurrence could be suspected at the same level or could have a different dermatomal distribution, in which case, a disc hernia at another level could be suspected. The differential diagnosis of a recurrent disc hernia is often difficult to establish because the disc recurrence can be associated with neurological manifestations specific to other pathological conditions: lumbar stenosis, segmental instability, peridural fibrosis. A relative clinic clue of the existence of a segmental instability is the improvement of the lumbar pains when the patient wears a lumbar belt. The most informative diagnostic methods of the disc recurrences, accompanied or not by segmental instability, are represented by MRI, MRI with contrast agent, MRI-myelography, CT-RSG, lateral dynamic X-ray [**1**,**8**].

The approach of the surgical treatment is dictated by the data complex, which includes the anamnesis, clinical picture and clinical-imagistic correlation. A special attention must be given to the patients who present expressed lumbar pains. In this case, the patient must be evaluated in order to establish a segmental instability and the surgical treatment will include the fusion of the operated segment.

The aim of the study was the analysis of the results of the surgical treatment of the patients with recurrent disc hernias with the application of a transforaminal intervertebral fusion and repeated laminotomy with the purpose of pain and disability improvement.

## Material and Methods

The clinical-imagistic examination was performed and the results of the surgical treatment of a lot of 56 patients diagnosed with ipsilateral recurrent disc hernias has been analyzed. The main surgical interventions have been made in many medical centers in the country.

In the primary surgery, the interlaminar approach has been applied in 31 patients (55,4%), in 16 patients (28,6%) – hemilaminectomy, an in 9 (16,1% - laminectomy. 

The patients were exposed to a thorough neurologic examination to appreciate the dynamics of the neurological signs compared to the main surgery. The basic neuroimagistic examination method was the contrast MRI, which has allowed the differentiation between the scar and the peridural fibrosis and a true disc recurrence. MRI myelography was done in 50 patients (89,3%).

The use of a contrast agent has significantly modified the algorithm and the treatment tactic of the patients with postoperative failures. Due to this investigation, it has become possible to identify and exclude the necessity of repeated surgery of patients with peridural fibrosis, who traditionally manifest the weakest results. In the same time, some studies showed that the postoperative success in ipsilateral recurrences at the same level, which were confirmed by contrast MRI, tend to get closer to the postoperative success of primary operations.

The process of forming of a peridural scar lasts for 3-4 months and the final organization takes place 6 months after the surgery. The internal organization of the intervertebral disc and the healing of the fibrous ring take place in the same period of time. The mature scar tissue is very well vascularized by a fine capillary network. The administration of the contrast agent leads to an increase of its concentration in the scar and the presence of a better signal in T1w. The MRI scanning was realized 15 minutes after the administration of “Magnevist” contrast agent in a concentration of 0,3mmols/kg through the administration of 7,5ml of contrast agent intravenously. The 15 minutes time interval after the administration of contrast agent in the scar is maximum, and, in the pulposus nucleus, it is practically absent. In this case, the recurrent disc hernia is manifested as a hypointensity area surrounded by a “capsule” which captures the contrast agent.

In turn, the pulposus nucleus starts to contrast to the 30th minute after the administration of “Magnevist” contrast agent. The presence of some areas surrounded by hypersignal zones is characteristic for the recurrent disc fragments or the residual disc fragments of scar tissue. The repeated surgery was realized at an interval of at least a year after the previous surgery, with a time interval of 1 to 3 years. The patients who presented clinical and radiological signs of segmentary instability associated with foot pain have been obligatorily evaluated to determine the radiological instability. Moreover, all the patients with expressed lumbar pains, accompanied by foot pains have been subjected to lateral dynamic MRI. This way, 30 patients with recurrent disc hernias without segmentary instability symptoms, and 26 patients with clinical-radiological symptoms characteristic to segmentary instability, have been identified.

The surgical technique was modified according to the changes that took place in the spinal canal after a discectomy surgery. When the decompression without fusion was planned, the surgery was done by skin incision on the line of the old scar with a blunt muscle detachment to the lamina. In case the lamina lacked, the incision and the muscle detachment have been easily extended superiorly to the base of the superior lamina. Usually, the repeated approaches are accompanied by more extended bone resections, which are conditioned by the necessity of identifying a segment without a scar and saved anatomical reports. The identification of lamina backlogs was done at the base of the spinous apophysis, closer to the medium line. An initial delimitation of the edge of the lamina and of the subjacent dural sac was done by dissector and Kerrison 1 bone punch. The dissection and discectomy stages were totally realized with the microscope. After the identification of the lamina edge, a lateral-superior resection of the obliquely oriented lamina was realized, through this obtaining a better exposure of the disc space and a larger mobility space for the execution of the maneuver. This moment is very important especially in cases of repeated surgeries due to the reduced mobility caused by the existent peridural fibrosis. The lateral-superior extension of laminotomy, associated with additional foraminotomy, allowed the obtaining of an adequate exposure of recurrent hernia, the nervous root and the disc space, without compromising the stability of the zygapophysial joint. A special attention was given to the lateral extension of the resection in order not to overcome 50% of the width of inferior joint apophysis.

Transforaminal lumbar intervertebral fusion (TLIF) was applied in a lot of 26 patients. The transforaminal approach was done both on the line of the old scar and through paramedian incisions together with an approach through Wiltse space. The transforaminal approach has a series of advantages, which are very important especially in repeated surgeries. The transmuscular access offers an operative way, without scars and risk of lesion of the dura, the angle of access towards the vertebral pedicle being of approximately 15 degrees, which is comfortable to place the transpedicular screws at level L3-S1. In case the presence of a massive peridural scar is noticed, the transforaminal approach can be realized by starting with the external part of the zygapophysial joint. This allows the precocious identification of the nervous root and its protection during the bone resection, with the purpose of decompression. In order to obtain a better exposure of the disc space and to avoid traction, the bone resection was completely done from one pedicle to the other, with the complete liberation of the neural foramen. An inferior resection of the sacrum was needed at level L5-S1 in order to have a comfortable access angle to disc L5-S1.

**Fig. 1:**
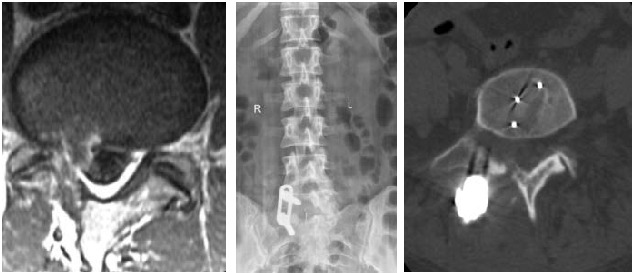
Recurrent disc hernia of L5-S1, on the right side a. imagistic aspect (contrast MRI); b. postoperative MRI – unilateral TLIF with a cage in PEEK; c. postoperative CT, axial aspect

Discectomy was done only after a complete decompression and the sufficient mobilization of the root, with the purpose of avoiding an avulsion of the root caught in the scar. The intervertebral fusion was realized with a “banana-shape” cage in the PEEK, placed in the 1/3 anterior side of the intervertebral space. The cage in the PEEK, but also the intervertebral space was filled with EquivaBone osteoinductive material to stimulate the fusion. “Legacy 5.5” (Medtronic, USA) transpedicular stabilization system with screws and rods was used for fusion. The ipsilateral TLIF imagistic results in a patient with disc recurrence with segmentary instability are presented in **Fig. 1**.

**Fig. 2:**
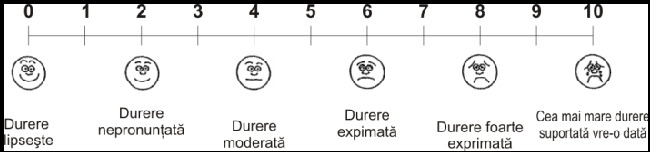
The visual-analogue scale of pain

The patients have been repeatedly examined at 3, 6 and 12 months postoperatively, then only once a year. The dynamic of the painful syndrome was underlined by using the visual analogue scale of pain (**Fig. 2**). An independent examiner analyzed the final postoperatory success at 1-3 years after the surgery, according to MacNab scale (**Table 1**) [**18**]. The functional disability was highlighted by the Oswestry Disability Index (ODI) scale ver. 2.1. (**Table 2**) [**16**,**17**].

**Table 1:** Modified MacNab scale of highlighting the postoperative results

Excellent	Without important pains. Without activities restrictions. Going back to the previous thing or the previous activity level.
Good	Occasional non-radicular pains that can be controlled by anti-inflammatory remedies. The patient is apt for work, but with certain modifications in the working regimen.
Moderate	A functional modest improvement; the patient is inapt for work, the presence of the handicap or the impossibility to enjoy the recreation activities due to severe intermittent pains.
Unsatisfying	Objective signs of radicular implication. Without an amelioration or an insufficient amelioration to improve the working activities or the activities regarding the social life. Necessity of an evaluation and repeated surgical treatment.

**Table 2:** The interpretation of the score obtained as a result of the evaluation, according to Oswestry social and professional disability scale

**Obtained score**	**Interpretation**
**0% - 20%** Minimum disability	The patients can cope with all the usual daily activities. No special treatment is needed, only limitations imposed at lifting weights and in doing exercises.
**21% - 40%** Moderate disability	The patients have serious problems in walking, sitting or in maintaining a vertical position for a longer period of time. The capacity of travelling or having a social life is possible but with significant difficulties. Some patients are not able to work (according to the specific of the working activities). Personal care, sexual life and sleep are not severely affected, which allows the maintenance of the satisfactory stage through non-operatory treatments.
**41% - 60%** Severe disability	The pain persistence represents the main problem in this group of patients and, this pain affects even the daily basic activities. A thorough evaluation for the decision regarding the further treatment graphic is needed.
**61% - 80%** Infirmity	Lumbar pain, located in the leg highly affects all the aspects of the life of the patient. Active invasive measures are needed.
**81% - 100%**	The patients are bed-ridden or they simulate

## Results and Discussions 

The accusations and the clinical manifestations that have led to the repeated addressing to medical care services are highlighted in **Table 3**. All the patients had different expression degrees of pains in the foot. In a lot of patients, the pain in the foot was accompanied by pains in the back. Most of the patients experienced differently expressed neurological disorders, and, 14 patients (25%) manifested signs of incomplete cauda equina syndrome.

**Table 3:** Patients’ clinical manifestations, n (%)

Clinical manifestations	Patients
Chronic pains in the foot (accompanied or not by lumbar pains)	56 (100%)
Cauda equina syndrome	14 (25%)
Sensitivity disorders	48 (85,7%)
Motor deficit	17 (30,4%)
Neurogenic claudication	39 (69,6%)

Contrast MRI represents the main investigation that allowed the identification of the cause of neurologic deficit in most of the cases, especially in patients previously operated. The patients who experienced important lumbar pains were recommended to undergo lateral dynamic lumbar MRI.

Beside the recurrent disc hernia, which was identified in all the cases, in 10 patients (17,9%), the disc recurrence was accompanied by a high degree of peridural fibrosis, and, in other 15 patients (26,8%), the extrusion of the disc fragments took place due to the presence of a lumbar canal stenosis, which was not solved by the surgery itself or was recurrent at the operated level. This way, all the cases presented a neuronal compression and a disco-radicular conflict, which represented the target of microsurgery manipulations.

The clinical and radiological segmentary instability signs have been detected in 26 patients. All these patients were subjected to a decompression treatment followed by a transforaminal intervertebral fusion. Out of 26 patients, in 20, the TLIF procedure was followed by bilateral transpedicular stabilization and 6 benefited from a unilateral stabilization due to symptoms predomination.

The pain expression degree was highlighted by using the visual-analogue scale of pain. It was noticed that the efficiency of pain amelioration in foot and back easily decreases according to the postoperative examined period. In addition, at 6 months postoperatively, a medium expression of 1,7-1,9 points of pain was highlighted according to the AVD scale, while, at 1 year postoperatively, the number has risen even more, to 2,2-2,4 points.

One of the criteria underlined was the sufferance period after the main surgery. It was noticed that the patients who underwent surgery in 6-12 months after the main surgery, showed results superior to the group of patients who had a sufferance period of 2 years and even more (80,4% vs. 37,5% according to MacNab scale) p<0,05. These data were also confirmed in the case of evaluating the patients by using Oswestry (ODI) functional disabilities appreciation questionnaire - (73,2% vs. 21,4%) patients with minimum disabilities at 6-12 months vs. >24 months after the first surgery.

The brief data obtained showed that, generally, the repeated surgical treatment proves a high degree success rate in ipsilateral disc recurrences. This efficiency was greater in the case of the treatment by transforaminal intervertebral fusion (70% vs. 76,9%), a fact that is explained through a wider transforaminal approach, a better decompression possibility, a reduced manipulation of the neuronal structures and a reduced risk of dura opening.

The main condition that assures the postoperative success is represented by the removal of all the neuronal compression factors with the maximal preservation of integrity of the posterior support elements of the spine in case of solitary decompression and the correct technical realization of the transforaminal fusion in cases of stabilization surgeries. These objectives were possible in the case of clinical neurological rigorous postoperative examinations and of contemporary neuroimagistic examinations. The perfect surgical technique, the thorough hemostasis and the decompression adequate to the neuronal structures, can all assure a clinically satisfying result of the patients who deal with a failure after a lumbar discectomy.

## Conclusion

The surgery repeated due to an initial postoperative failure needs a rigorous clinical-imagistic evaluation, which should also include the contrast MRI, which allows the differentiation between a disc hernia recurrence and the peridural fibrosis. Superior-lateral discectomy through laminotomy represents a surgical measure sufficient for the cases of disc hernia recurrence without instability signs. The supplementation of radicular decompression with a transforaminal intervertebral fusion is argued from a physiopathological point of view in the cases in which there are clinical or segmentary instability radio-imagistic signs. According to Oswestry and MacNab evaluation scales, the best results can be obtained in cases of surgeries that take place in a time interval of at most 12 months after the main surgery.
